# Correction: Electroencephalography as a diagnostic tool for late-onset efavirenz neurotoxicity syndrome

**DOI:** 10.1371/journal.pone.0339300

**Published:** 2025-12-18

**Authors:** Sam Nightingale, Salvatore Ssemmanda, Lawrence M. Tucker, Roland W. Eastman, Eddy B. Lee Pan

Fig 1 was uploaded incorrectly. Please see the correct Fig 1 here.

**Fig 1:**
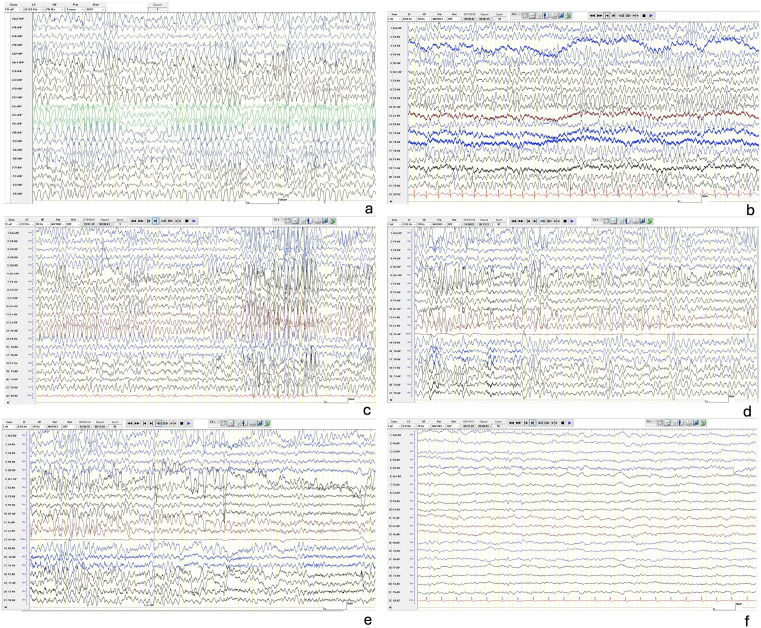
Characteristic EEG changes of LENS. a-e are 5 EEG epochs from 4 LENS cases showing persistent, diffuse, high voltage, bisynchronous, monomorphic 4–7 Hz theta frequency waveforms. Transient attenuation on eye opening is shown in e (same case as d). f is a non-LENS control for comparison—a 44 year-old male living with HIV with disseminated TB and acute hydrocephalus. EEG shows some low amplitude semi-rhythmic theta but in contrast to LENS this is intermittent and varying, mixed with low amplitude fast frequencies and some low amplitude delta transients. 3 further examples from non-LENS controls are given in supplementary material.
