# Supplementary figures and images for: Electroencephalography as a diagnostic tool for late-onset efavirenz neurotoxicity syndrome
Source: PLoS One. 2023 Nov 10;18(11):e0288055. doi: 10.1371/journal.pone.0288055 (PMC10637668; doi:10.1371/journal.pone.0288055)

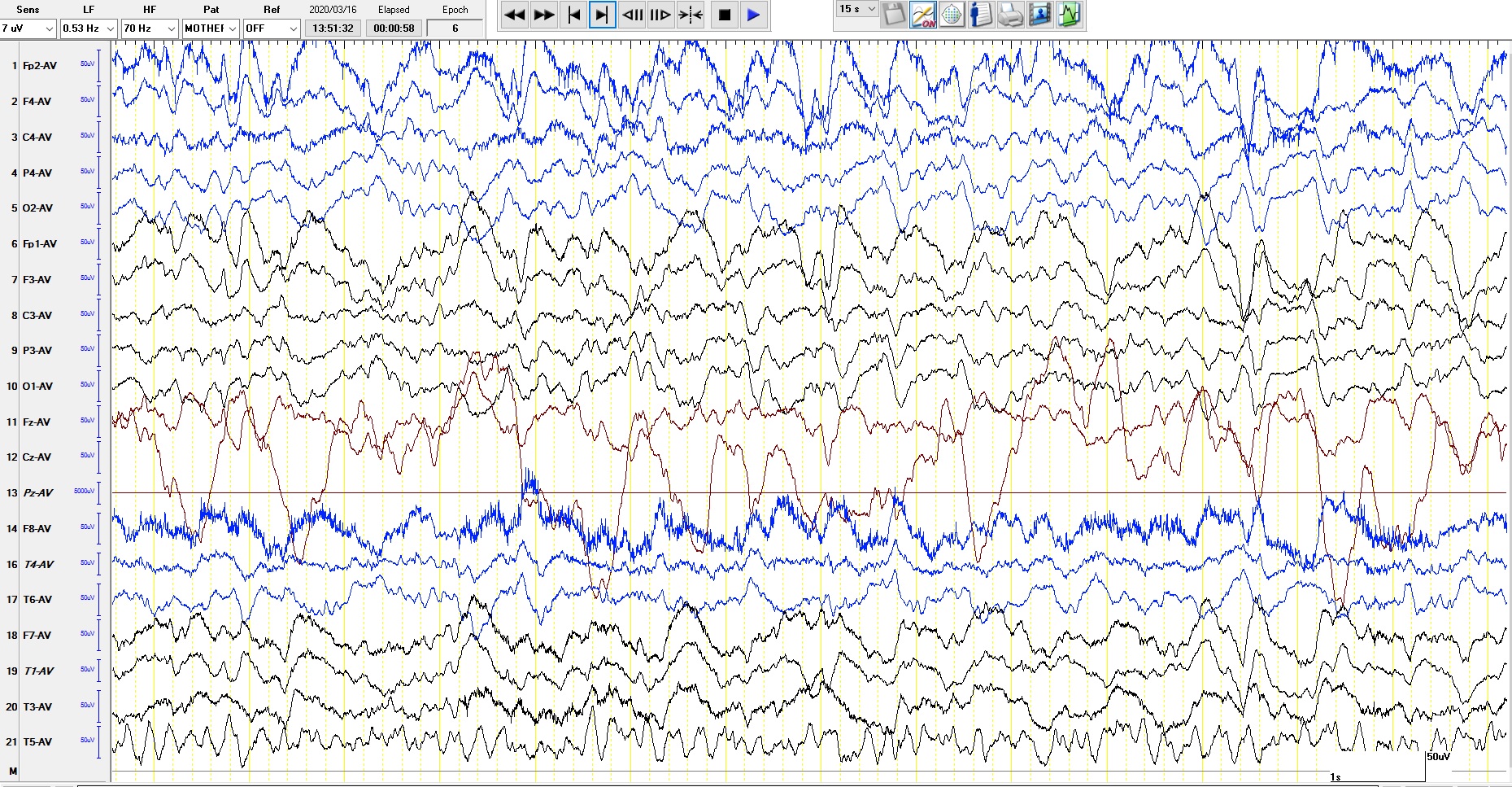

Supplement: S1 Fig — a. 58 year-old woman living with HIV. Diagnosis: Wernicke’s encephalopathy. EEG shows a mixed background with large theta and delta frequencies, with superimposed semi-rhythmic theta and faster frequencies. In contract to LENS cases, the semi-rhythmic activity is unevenly distributed in time and space and generally low amplitude. b. 32 year-old HIV-negative female. Diagnosis: acute disseminated encephalomyelitis (ADEM). EEG was marred by excessive EMG artefacts. Epoch demonstrated has filtered the high frequencies to show the underlying brain activity, although some residual EMG attracts are still evident. There is intermittent theta but this contrasts to LENS cases due to the presence of a mixture of other frequencies with delta transients, fragments of alpha and also some beta activity. c. 47 year-old HIV-negative female. Diagnosis: hepatic encephalopathy. EEG shows quite rhythmic fast theta sinusoidal activity which fluctuates between low and very low amplitudes. Some low amplitude faster frequencies also present and occasional slower theta transients (the latter not shown in this epoch). Of the 4 control EEGs shown here, this would be the most similar to LENS cases, but lacks the very high amplitudes associated with this condition. (ZIP) [file pone.0288055.s001.zip › Figure 2a_EEG for LENS_June 2023.jpg]

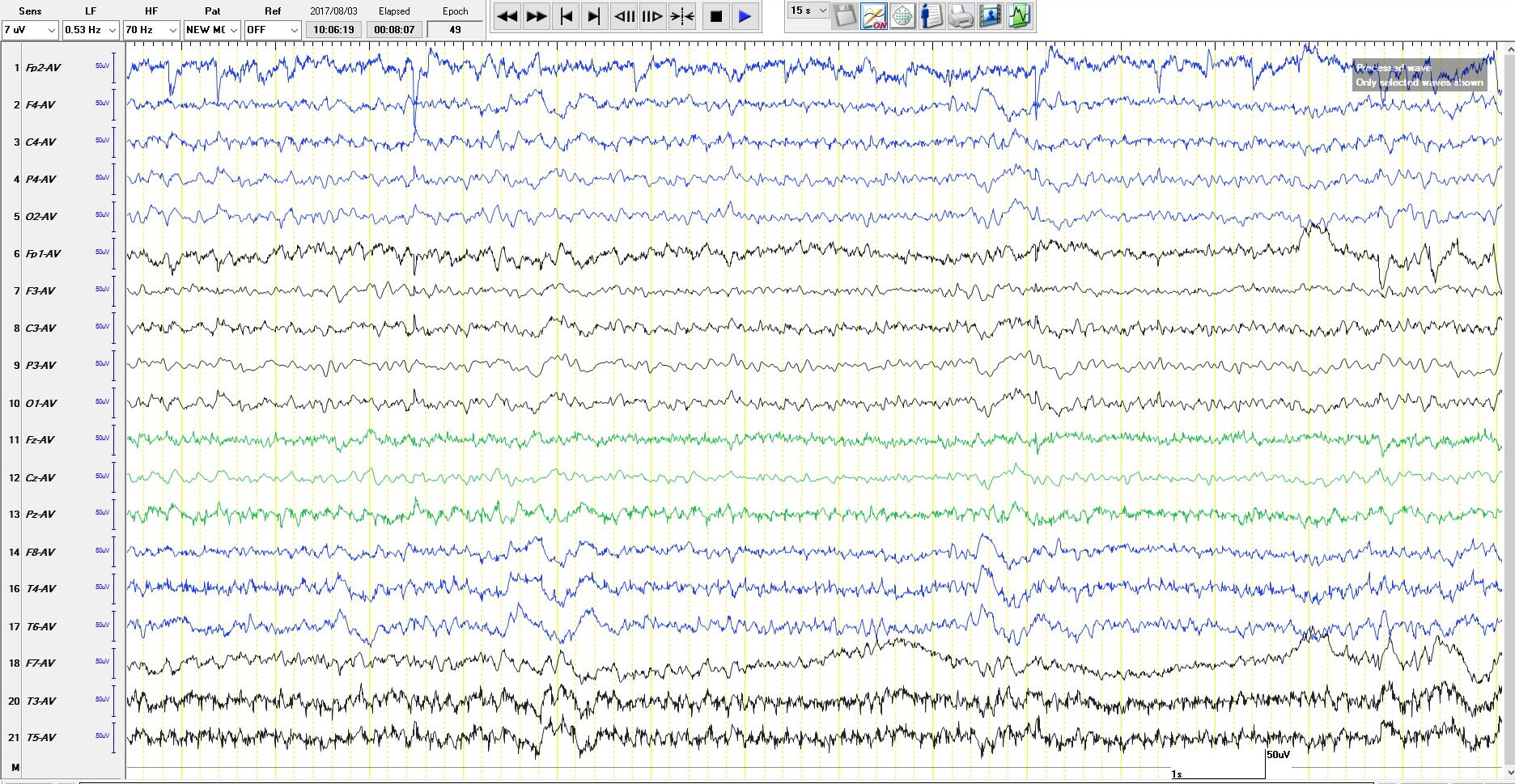

Supplement: S1 Fig — a. 58 year-old woman living with HIV. Diagnosis: Wernicke’s encephalopathy. EEG shows a mixed background with large theta and delta frequencies, with superimposed semi-rhythmic theta and faster frequencies. In contract to LENS cases, the semi-rhythmic activity is unevenly distributed in time and space and generally low amplitude. b. 32 year-old HIV-negative female. Diagnosis: acute disseminated encephalomyelitis (ADEM). EEG was marred by excessive EMG artefacts. Epoch demonstrated has filtered the high frequencies to show the underlying brain activity, although some residual EMG attracts are still evident. There is intermittent theta but this contrasts to LENS cases due to the presence of a mixture of other frequencies with delta transients, fragments of alpha and also some beta activity. c. 47 year-old HIV-negative female. Diagnosis: hepatic encephalopathy. EEG shows quite rhythmic fast theta sinusoidal activity which fluctuates between low and very low amplitudes. Some low amplitude faster frequencies also present and occasional slower theta transients (the latter not shown in this epoch). Of the 4 control EEGs shown here, this would be the most similar to LENS cases, but lacks the very high amplitudes associated with this condition. (ZIP) [file pone.0288055.s001.zip › Figure 2b_EEG for LENS_June 2023.jpeg]

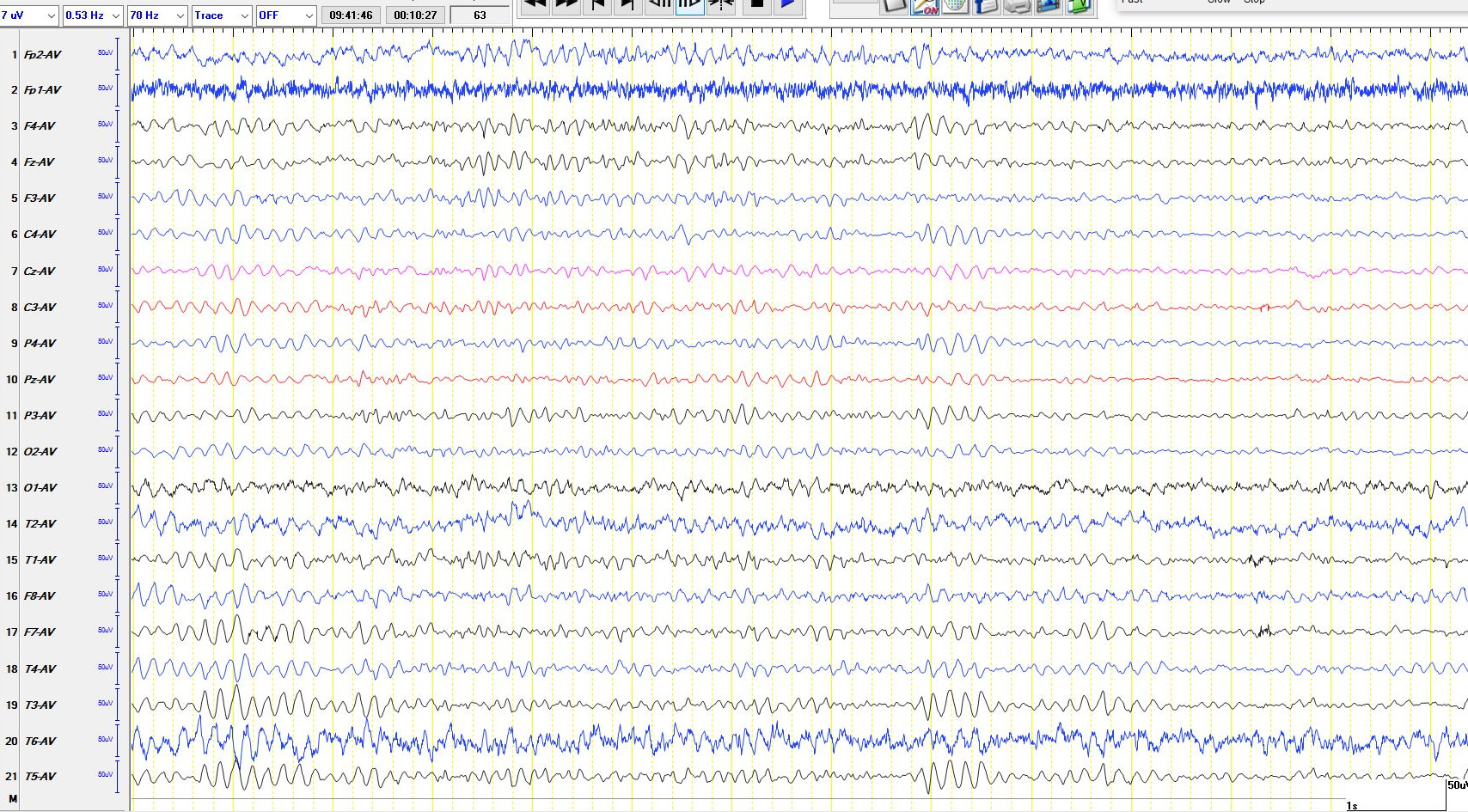

Supplement: S1 Fig — a. 58 year-old woman living with HIV. Diagnosis: Wernicke’s encephalopathy. EEG shows a mixed background with large theta and delta frequencies, with superimposed semi-rhythmic theta and faster frequencies. In contract to LENS cases, the semi-rhythmic activity is unevenly distributed in time and space and generally low amplitude. b. 32 year-old HIV-negative female. Diagnosis: acute disseminated encephalomyelitis (ADEM). EEG was marred by excessive EMG artefacts. Epoch demonstrated has filtered the high frequencies to show the underlying brain activity, although some residual EMG attracts are still evident. There is intermittent theta but this contrasts to LENS cases due to the presence of a mixture of other frequencies with delta transients, fragments of alpha and also some beta activity. c. 47 year-old HIV-negative female. Diagnosis: hepatic encephalopathy. EEG shows quite rhythmic fast theta sinusoidal activity which fluctuates between low and very low amplitudes. Some low amplitude faster frequencies also present and occasional slower theta transients (the latter not shown in this epoch). Of the 4 control EEGs shown here, this would be the most similar to LENS cases, but lacks the very high amplitudes associated with this condition. (ZIP) [file pone.0288055.s001.zip › Figure 2c_EEG for LENS_June 2023.jpeg]
