# Supplementary material for: Electroencephalography as a diagnostic tool for late-onset efavirenz neurotoxicity syndrome
Source: PLoS One. 2023 Nov 10;18(11):e0288055. doi: 10.1371/journal.pone.0288055 (PMC10637668; doi:10.1371/journal.pone.0288055)
Supplement: Attachment — Submitted filename: Reviewer response EEG LENS_14 June 2023.docx [file pone.0288055.s003.docx]

**Response to reviewers’ comments – 14 June 2023:**

**Electroencephalography as a diagnostic tool for late-onset efavirenz neurotoxicity syndrome**

*Additional Editor Comments:
Please provide more EEG traces. It would be useful for the reader if at least 3 EEG of patients affected by LENS are included in the paper, given the rarity of this disease. Inclusion of slight different EEG traces will improve the skills also of our readers.
As reference, provide at least 1 (and 3 more as supplementary material) EEG traces of control patients, describing their diagnosis and EEG findings.*

**The editor requested more examples of LENS EEGS, and some examples of control EEGs, with diagnosis and description. We have now provided those – see figure 1 and supplementary material.**

**The minimum dataset has been added as a supplementary file**

*Reviewer #1: The authors examined EEG as a diagnostic tool for LENS. To do so, they conducted a retrospective case-control study including 13 LENS cases and 50 controls. Two blinded interpreters examined the EEGs. Interpreter 1 labeled LENS cases 11/13 as having definite or probable changes, while interpreter 2 labeled 10/13. Interpreter 1 labeled probable changes in controls 1/50 and interpreter 2 3/50. Interrater reliability was good and the sensitivity of the EEG for the diagnosis of LENS was around 80% and the specificity was over 90%. The authors conclude that EEG is a useful tool in the diagnosis of LENS.
I have no comments*

**No specific issues to respond to from reviewer 1.**

*Reviewer #2: This is a retrospective case-control study aimed to compare EEGs from 13 confirmed cases of late-onset
efavirenz (EFV) neurotoxicity syndrome (LENS) with 50 control EEGs recorded in adults with a similar indication for EEG, dysrhythmia generalised grade II, and without LENS. Interrater (1 and 2) reliability was adequate, sensitivity of EEG for the diagnosis of LENS was 85% and 77% for interpreters 1 and 2 respectively, and specificity was 98% and 94%. So the Authors demonstrated that EEG is a useful and cheap tool in the diagnosis of LENS, before to get the EFV level.
Data are clearly reported and discussed. Statistical analysis is adequate. The findings are relevant and helpful to address the daily clinical practice with patients presenting LENS.*

**No specific issues to respond to from reviewer 2.**

**Further to above, we noticed a minor error in table 1. The mean age given in the ‘controls’ column was in fact the mean age of the cohort. We have corrected this - it does not materially alter the paper in any way.**
